# Unknotting the interactive effects of learning processes on cultural evolutionary dynamics

**DOI:** 10.1017/ehs.2019.17

**Published:** 2019-12-23

**Authors:** Lauren A. Scanlon, Andrew Lobb, Jamshid J. Tehrani, Jeremy R. Kendal

**Affiliations:** 1 Department Of Mathematics, Durham University, Durham, UK; 2 Department Of Anthropology And Durham Cultural Evolution Research Centre, Durham University, Durham, UK; 3 Durham Research Methods Centre

**Keywords:** Approximate Bayesian computation, copying error, cultural evolution, knots, social learning

## Abstract

Forms of non-random copying error provide sources of inherited variation yet their effects on cultural evolutionary dynamics are poorly understood. Focusing on variation in granny and reef knot forms, we present a mathematical model that specifies how these variant frequencies are affected by non-linear interactions between copying fidelity, mirroring, handedness and repetition biases. Experiments on adult humans allowed these effects to be estimated using approximate Bayesian computation and the model is iterated to explain the prevalence of granny over reef knots in the wild. Our study system also serves to show conditions under which copying fidelity drives heterogeneity in cultural variants at equilibrium, and that interaction between unbiased forms of copying error can skew cultural variation.

## Graphical abstract

**Figure:**
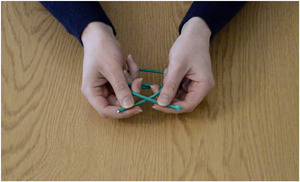

**Media Summary**: We statistically identify forms of copying error when tying simple knots, and show how they may interact to affect knot evolution.

## Introduction

Scholars studying the evolution of human tools have noted that forms of copying error may affect variation in the manufactured forms. For instance, computational and experimental simulation studies show how random copying error that is imperceptible to the learner can result in amplified population-level variation over generations (Eerkens and Lipo, 2005; Kempe et al., 2012). However, the relationship between variation in behaviour and the resultant variation in artefacts is not necessarily linear. By mathematical derivation, Hamilton and Buchanan (2009) show that if the magnitude of normally distributed copying error is proportional to the copied object, variation in artefact design evolves through geometric Brownian motion and the mean of the artefact distribution drifts to the left. Thus random copying error can have non-random evolutionary consequences.

While unbiased, normally distributed error can affect evolutionary dynamics, it is plausible that forms of copying error may be non-random and that the accumulation of these errors over generations can affect artefact variation. For example, Kempe et al. (2012) provide putative evidence that artefact size may increase or decrease over generations of reproduction depending on whether the object is constructed by reductive or additive techniques, respectively. Multiple forms of copying error may contribute to artefact construction. For instance, if the learner has to copy a bilaterally symmetrical action, they may attempt the mirror image of the demonstrated action (an instantiation of the correspondence problem; Heyes and Bird 2007) and, in addition, their choice of action could be affected by their own handedness (Laland et al., 1995). If the behaviour includes a sequence of actions, the learner may be inclined to repeat the action they just performed over copying a similar but different action. These forms of copying error may apply across a wide variety of contexts, from motor patterns in tool production, writing, painting and sculpture to athletic activities such as dance or sporting techniques.

Our project concerns the effects of copying errors on the cultural evolution of knot tying. Ashley (1993) collated over 3800 examples of knots used for a wide variety of functions, ranging from the simple overhand knot which is characterised by only three crossing points, to extremely complex knots with at least 16 crossings. Nonetheless, only a small proportion of all possible knots are actually used and it is unlikely that only these knots could satisfy functional requirements (Scanlon, 2016). Thus the observed variation may have been contingent not only on functional sufficiency but also on modes of social transmission, perceived risk of modifying a knot, the use of knot structures as symbolic markers and learning processes that result in systematic copying errors.

Our study focuses on this latter effect for a family of simple knots which are the overhand knots. These knots can take different forms, characterised by their handedness, which, despite their simplicity, are susceptible to being copied incorrectly. Systematic copying errors across generations of learners may have affected variation not only in these simple knots, but also in more complex knots which include overhand knot structures.

**Figure 1.**
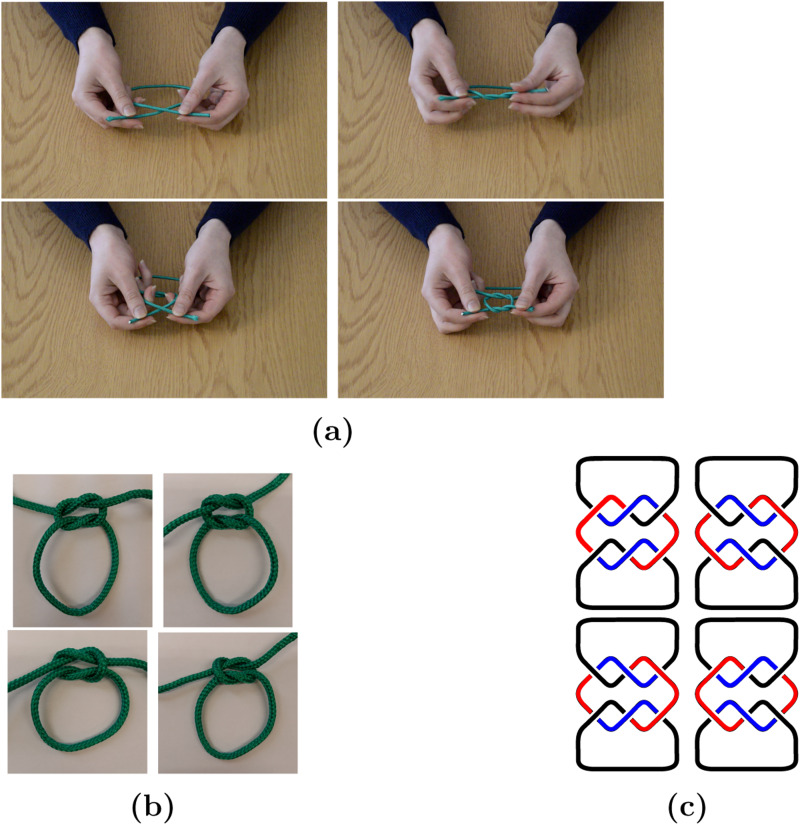
(a) Screenshots from a demonstration video used in the experiment, (b) tied versions of all four knots used and (c) the four possible combinations of overhand knots depicted as three-dimensional closed curves. Parts (b) and (c) both show: top left, LL granny knot; top right, RR granny knot; bottom left, LR reef knot; bottom right, RL reef knot.

The composition of overhand knots (Alexander–Briggs notation: 3_1_#3_1_) is formed from two trefoil knots (A–B notation: 3_1_), each of which is tied by feeding one end of a string through a loop and can take either a right- (R) or left-handed (L) form (Figure 1). The composition of two left- or right-handed trefoils is commonly known as a granny knot (LL or RR), while the compositions of a left- and a right-handed trefoil are classed as reef knots (LR or RL). Originating approximately 300,000 BP and preserved from 5500 BP (Van der Kleij, 1998; Warner and Bednarik, 1998), these knots are a relatively ubiquitous technology (Ashley, 1993). A common use of the composition of overhand knots is to tie shoelaces with an overhand knot followed by a slipped overhand knot on top, although the motor and visual patterns used to preserve the loops in the shoelaces differ from those associated with simply tying a generic composition of overhand knots: tying one trefoil after another without a loop. Analysis of the *Ashley Book of Knots* (1993) revealed that of the wide variety of knots containing granny and reef structures, the granny knot appears in 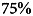 of cases (Scanlon, 2016). An analysis of impact in the integrity of knot structure shows that the reef knot is less liable to come undone, suggesting that non-functional biases may be required to explain the prevalence of the granny over the reef forms (Grog, n.d.; O'Reilly et al., 2017).

Mathematically, a knot is a three-dimensional closed curve, where the string is over and under itself in some way with the ends glued together. The left- and right-handed versions of the trefoil are mirror images of one another and are mathematically distinct as they cannot be transformed into each other by Reidemeister moves, a set of moves on the strands of a knot used to determine if two diagrams relate to the same knot (Reidemeister, 1927); the only way to change the left-handed trefoil to the right-handed trefoil is to cut the knot open and retie it. The granny and reef knots are distinct knots, and can be identified as such by knot invariants (Adams 2004; see Supplementary Material Section S1). The granny knots are distinct from each other and both reef knots, but the two reef knots are not distinct, which can be seen by rotating one reef knot to match the other; no such rotation is possible for the granny knots (Figure 1c).

Our study uses social transmission data to explore the interactive effects of learning processes on cultural evolutionary dynamics. We ran a social learning experiment whereupon participants were exposed to demonstration of one of the four composition of overhand knot variants and asked to copy the observed knot. This generated data on the change in frequencies of the four knot variants across sequential generations of knot tyers. We then present a model that describes the non-linear, interactive effects of four putative learning processes (copying fidelity of the perceived demonstration, mirroring, handedness bias and repetition) on the change in the composition of overhand knot frequencies across sequential generations of knot tyers. We explore cultural evolutionary dynamics of the system before applying approximate Bayesian computation (ABC; Kandler and Powell 2018) to derive posterior estimates for the four learning processes. These estimates were used to predict evolutionary trajectories of the four knot variants which can then be compared against what we know of granny and reef knot frequencies in the wild.

## Social transmission experiment

Participants were recruited from the student population of Durham University. They were rewarded with a *£*4 food voucher for their participation. In total 101 people took part in the experiment with 36 males. None of the participants were experts at learning to tie knots on command. Similar to adult learners in the wild who may attempt to copy the demonstration of the knot structure, the participants typically had some prior experience tying simple knots, although approximately two-thirds claimed they did not know how to tie either a granny knot or a reef knot (see Section S3), and some even claimed to be unable to tie shoelaces.

The experiment took place in a lecture theatre, with batches of up to 10 participants at a time. We treated between-participant effects as independent by spacing participants widely across the lecture theatre and requiring each participant to tie their knots within a modified cardboard box, which prevented between-participant observation.

For the social transmission experiment, participants were given a 35cm length of string and, using the overhead projector, shown a video demonstrating the tying of an LL granny knot (26 participants), an RR granny knot (25 participants), an LR reef knot (25 participants) or an RL reef knot (25 participants), randomly assigned across batches. Screenshots of the video and the knots shown in Figure 1a clarify that the demonstrated knot was tied in its generic form, without the loops typically retained when tying shoelaces. Participants were instructed that the aim was to copy the knot shown in the video, which showed only hands tying a knot and contained no audio. The video was recorded from the point of view of an observer sitting opposite the demonstrator, so the observer would have to take the demonstrator's perspective to copy the correct knot handedness. Participants were shown the video three times, with a pause of 30 seconds between each showing. They were told they could practice tying the knot whilst the video was being shown, and during the pauses between the showings. After the final showing of the video, they were told to untie any practice knots and to tie the knot shown in the video. Of the 101 knots tied after being shown the video, 100 of the knots were either LL, RR, LR or RL, and the remaining knot (a composition of the double overhand knot, 5_1_in the Alexander–Briggs notation, and the trefoil knot) was excluded from the analysis.

In additional exploratative analysis, described in Sections S2 and S3, we performed an asocial test of each participant's handedness bias run prior to the social learning experiment, and administered a short questionnaire after the social learning experiment requesting the participant's name, gender, degree programme, handedness, hand usually used for writing, and whether they knew how to tie a granny or reef knot.

Table 1 indicates that participant behaviour in the social learning experiment was affected by the demonstration they observed. They were most likely to tie the knot shown in the video (the leading diagonal), but if a mistake was made, participants were most likely to tie the mirror image of the demonstrated knot over the other two variants. For example, more people tied the RR granny knot when shown LL than tied the reef knot, LR or RL. Also, granny knots were more likely to be tied than reef knots, suggesting that participants may exhibit a bias to repeat the handedness of the first trefoil they tie. Finally, there was a very small bias towards left- over right-handed knots in the sample. See Section S4 for probability distributions of each knot being tied in response to a given demonstrated knot and for associations with trefoil handedness bias under asocial conditions (Section S2) and the questionnaire results (Section S3).

**Table 1.** Knots tied by participants given video shown in the experiment; dashed lines delineate granny knots from reef knots

		Knot tied by participants				
		LL	RR	LR	RL	Total
Demonstration	LL	14	9	1	2	26
	RR	9	15	0	1	25
	LR	4	4	8	8	24
	RL	6	1	6	12	25
	Total	33	29	15	23	10

We suspect that multiple learning processes may be interacting to affect social transmission of the four variants so in the next section we identify four putative processes and specify in a model how they interact non-linearly to affect the social transmission of these knots. After exploring the cultural evolutionary properties of this model, we apply ABC to the experimental data, deriving posterior estimates and predicting evolutionary trajectories.

## Social transmission model

### Assumptions

We model the transmission of granny and reef knots within a population through oblique transmission (Cavalli-Sforza and Feldman, 1981) and assume a closed system such that when a granny or reef knot is demonstrated, the learned knot is always either a granny or a reef knot. We assume that four parameters can affect the fidelity of social transmission: the learner interprets the demonstrator's knot incorrectly as the knot's mirror image with a probability *g* (mirroring); the learner copies the perceived trefoil with a probability *s* (copying fidelity), where the perceived trefoil refers to the learner's interpretation of the demonstrated trefoil, which could either be the demonstrated knot or the mirror image of the demonstrated knot; the learner repeats the trefoil they tied for the first step of the composition of overhand knots with a probability *r* (repetition); and the learner ties a right-handed trefoil when they do not copy the perceived demonstration with a probability *p* (handedness).

Using these parameters, we can build a system of recurrence equations to describe relative knot frequencies in the learner generation as a function of their frequencies in the demonstrator generation. We denote the proportion of knot *ij* tied in the demonstrating generation by *f*_*ij*_ where *ij* ∈ {RR, LL, RL, LR}, and the knots tied by the learner generation of the population after transmission as 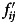 where 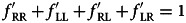 with each 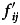 taking values in the interval [0,1]. For example, take the granny knot formed by tying two right-handed trefoils and denote it by *f*_RR_. This knot will be transmitted successfully if it is not mirrored and both trefoils that form it are accurately copied by the next generation, denoted by 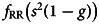. However, a right granny could also be formed by mirroring an LL with probability *g* and accurately copying both trefoils of the perceived knot with probability *s*^2^, giving *f*_LL_(*s*^2^*g*). A right granny could also be formed with no copying fidelity at all (*s* = 0), if the learner has a bias towards tying right-handed trefoils 
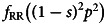
 or repeating the first knot tied, 
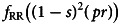
 and so we get the frequency of right granny knots in the population as a function of granny and reef knots already in the population and the probability parameters:1
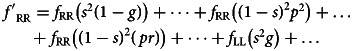


**Figure 2.**
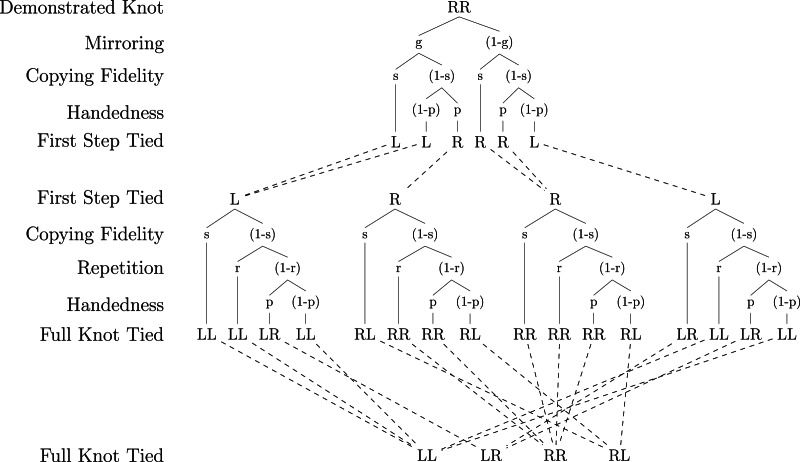
Probability tree showing the effect of parameters on the transmission of knot RR. For each trefoil tied, the order by which parameters are shown to take effect along a given branch is arbitrary as the probability that the knot is of form L or R is simply the product of their combined effect.

It is important to think about how the parameters interact with each other. If a learner copies the knot correctly then the learner's likelihood to repeat or tie a right-handed trefoil does not matter. They will do what is shown regardless of their handedness bias or propensity for repetition, and so we can discount repetition and right-hand bias when the knot is accurately copied. In the same way, when the learner simply repeats part of a knot, their right-hand bias does not matter, as they will repeat regardless of this bias. So we can discount right-hand bias when repetition takes place. Figure 2 illustrates how the knot tied may be affected by the observed knot and the four parameters. Note that for each trefoil, the depicted order of parameters on any given branch is arbitrary and does not affect the probability that a particular trefoil form is tied (i.e. the parameters commute; see S5). For instance, the first trefoil can be tied left-handed if the learner both fails to copy the perceived knot and is not subject to a right-handedness bias irrespective of any order by which these processes might take effect. For each trefoil, the model only accounts for combinations of learning processes that lie on the same branch of the probability tree (see Discussion).

### Evolutionary dynamics

Each set of parameter values 0 ≤ (*s*, *g*, *r*, *p*) ≤ 1, determines the evolutionary trajectory and a single equilibrium point, where 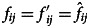, (expressions for equilibrium states are given in Section S6). If *s* = 0, the system jumps to a stable equilibrium point determined by the *p* and *r* and is unaffected by starting values of *f*_*ij*_. In contrast, if copying is always accurate, *s* = 1, and mirroring never occurs, *g* = 0 (0 ≤ *r* ≤ 1), the population does not evolve from starting frequencies, so if a small perturbation in frequencies is induced, the population remains at the new frequencies. If there is some copying, 0 <*s* <1, the population evolves to a stable equilibrium, such that the population returns to the original equilibrium state following a small perturbation in frequencies.

**Figure 3.**
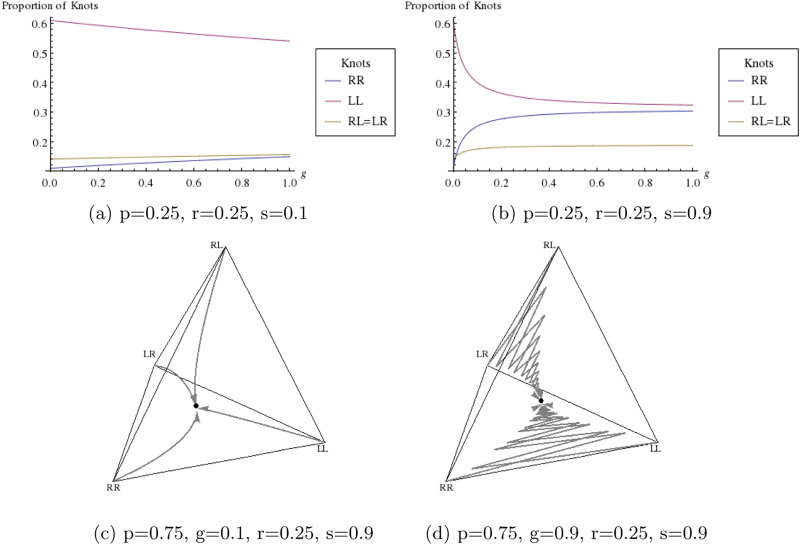
Parts (a) and (b) show the proportion of knots at equilibria as a function of the probability of mirroring when copying fidelity of the perceived knot is low and high, respectively. The values of 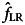 and 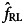 are equal so these are represented by the same line on the graph, while 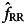 and 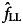 are represented by separate lines. Parts (c) and (d) show evolutionary trajectories when the probability of mirroring is low and high, respectively. Each arrow represents the change in relative frequency of each type of knot in the population, starting from sole existence in each corner to a mixture of different knots in the interior of the tetrahedron. The solid disc is the equilibrium state which is evolved towards no matter the starting frequencies. Frequencies are plotted in tetrahedral space using Barycentric coordinates (see Section S8).

Figure 3 illustrates the effect of copying and mirroring on equilibrium frequencies. In Figure 3a, the value of *s* is set lower than in Figure 3b, resulting in a relatively small change in the values of 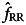, 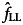 and 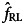 and 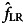. This shows that copying needs to be highly probable for mirroring to affect the proportion of knots tied in the population. We notice that the two reef knot frequencies, *f*_LR_ and *f*_RL_, are always equal at equilibria. This is consistent with the fact that LR and RL represent the same knot mathematically (see Section S1).

Prior to reaching an equilibrium state, evolutionary dynamics typically follow a smooth trajectory (assuming 0 < *s* < 1), but a high probability of mirroring can cause oscillations in the trajectory when copying fidelity is high. When mirroring is low (Figure 3c), we see the system evolve in a smooth curve to a point strongly affected by the handedness, *p*and repetition, *r*. The high value of *p* causes the point to be slightly closer to the corner *f*_RR_ = 1 than *f*_LL_ = 1 but the low value of *r* does not cause the point to be as close to the *f*_RL_ + *f*_LR_ = 1 boundary as we may expect. In Figure 3d, mirroring is likely to occur. Coupled with the high copying fidelity, the system evolves to a similar equilibrium point as shown in Figure 3c, but the high probability of mirroring causes the path to oscillate to the point rather than evolve in a smooth trajectory.

Humans are likely to copy a perceived demonstration with some success but to make some mistakes. In this circumstance (0 < *s* < 1), there are some conditions where *s* does not affect equilibrium state frequencies.

If *p* = 1/2, we have
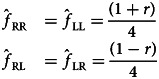
while if *g* = 0, we have
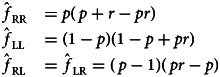


**Figure 4.**
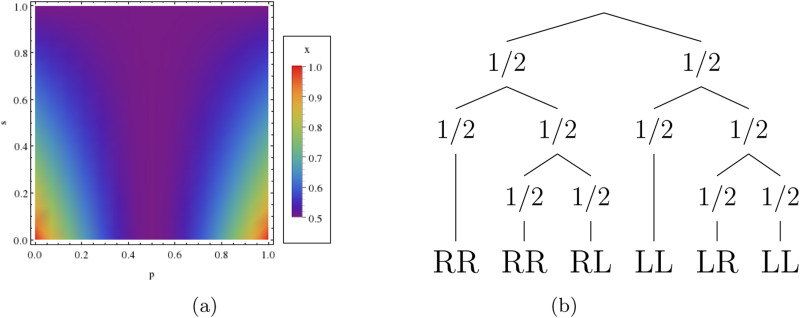
(a) A density plot showing the proportion of granny knots at equilibrium, denoted by 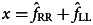, as a function of handedness bias, *p* and copying fidelity, *s*, where *g* = 1/2 and *r* = 0. (b) A probability tree showing knots tied in the absence of biases in handedness (*p* = 1/2; top two layers affecting first and second trefoil) and repetition biases (*r* = 1/2; bottom layer, affecting second trefoil).

Most combinations of parameter values result in an excess of granny knots over reef knots at equilibrium. As noted above, any repetition, *r*, will favour the granny knot, but even when repetition never occurs, the population is still more likely to tie granny knots than reef knots if there is any handedness bias, *p* ≠ 1/2. Mirroring typically has little influence on the relative equilibrium frequency of granny to reef knots when 0 < *s* < 1 and has no influence when either *s* = 0 or *s* = 1. Figure 4a illustrates the predominance of granny knots at equilibrium, taking the case where there is no repetition in the absence of guidance, *r* = 0, and intermediate mirroring, *g* = 1/2. The bias towards granny knots is strongest when handedness bias, *p*, is either high or low and the copying coefficient, *s*, is low; in other words, when individuals consistently tie with the same handedness rather than copying a different knot.

**Figure 5.**
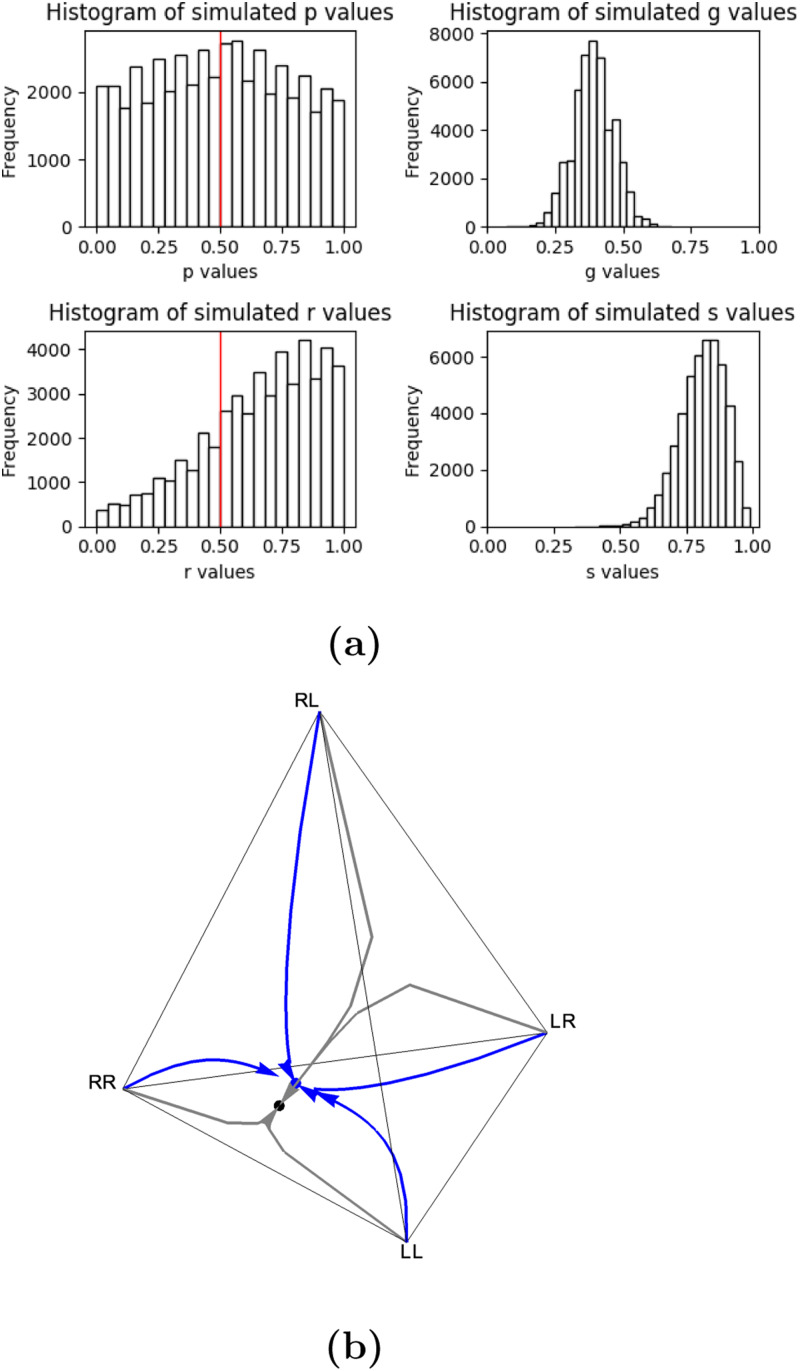
(a) Histograms of parameter values simulated from the experiment, with acceptance interval *d*(*O*,*S*) ≤ 0.0075. Red lines indicate unbiased parameter values, *p* = 1/2 and *r* = 1/2, giving equal probability of tying right- and left-handed trefoils and equal probability of repeating the previous knot as not, respectively. (b) Evolutionary trajectories of the four knot forms, where *f*_*ij*_ = 1 in each corner and frequencies are equal at the centre of the tetrahedron. Trajectories using the mean posterior parameter values 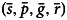 are shown by the grey arrows and black disc, 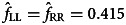, 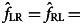 0.085. The blue arrows and disc, 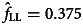, 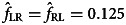, 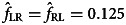 show the trajectories in the absence of handedness bias and repetition bias (*p* = 1/2, *r* = 1/2) assuming no mirroring, *g* = 0, and the mean posterior parameter value for copying fidelity, 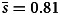 (note that mirroring and copying fidelity do not affect the equilibrium state here).

Also note that the absence of non-random copying error does not lead to equal knot frequencies; granny knots are expected in higher frequency than reef knots (Figure 4b; also see that in Figure 5b the blue disc is not in the centre of the tetrahedron). This occurs because of the way the parameters interact to affect the knot forms: consider for instance the case in the absence of handedness bias and repetition bias *p* = 1/2, *r* = 1/2 (under these conditions, mirroring and copying fidelity do not affect equilibrium frequencies). Figure 4b shows that the probability of tying each knot is *P*(LL) = 3/8, *P*(RR) = 3/8, *P*(RL) = 1/8 and *P*(LR) = 1/8.

There are only two cases where the equilibrium proportion of granny and reef knots is equal 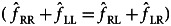. The first case is when copying is not perfect, 0 ≤ *s* < 1, the first knot is never repeated, *r* = 0, and there is no handedness bias, *p* = 1/2, where 0 ≤ *g* ≤ 1. The absence of repetition prevents predominance of granny knots, and the lack of handedness bias prevents the prevalence of either granny knot. The second case is when copying is perfect, *s* = 1, and there is some mirroring 0 < *g* ≤ 1, where 0 ≤ *p* ≤ 1 and 0 ≤ *r* ≤ 1. Here copying the perceived knot form is always perfect, but mirroring causes tying of the opposite handedness to that demonstrated. Both these cases are illustrated in Figure 4a. Finally, we note that reef knots can only be more prevalent than granny knots if this is exhibited in their starting frequencies and when the system does not evolve (*s* = 1 and *g* = 0; discussed above).

### Fitting the social transmission model to experimental data

Using ABC (Sunnåker et al. 2013), we can use our model to estimate parameter values that predict the experimental data. ABC works on the same premise as Bayes’ theorem, relating conditional probability of parameters *θ*, to data *D* by the rule2
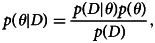
where *p*(*θ*|*D*) is referred to as the posterior, *p*(*θ*) represents the prior beliefs before any data is available, *p*(*D*|*θ*) the likelihood of data *D* occurring given the prior and *p*(*D*) the evidence (Gelman et al., 2003). With this rule, we can calculate the posterior by taking the product of prior beliefs with the likelihood of data occurring, divided by the evidence observed. To obtain the probability of data *D* given parameter *θ*, we use our model to simulate data for a given parameter set and decide whether it fits the observed data. We construct a metric to describe our observed data such that we can accept or reject the simulated parameter set depending on whether it generated data within a tolerated proximity from the observed. The retained parameter distributions give us *p*(*θ*|*D*).

Taking our observed data from Table 1 as a 4 × 4 matrix *O* and simulating data of the same form using our model to give a 4 × 4 matrix *s*, we compare these two sets of data using the metric;3
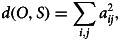
where *a*_*ij*_ are the entries of the matrix *O* − *S*. This metric is proportional to finding the Euclidean distance between the points in the two matrices.

Describing the process in more detail, we ran a Monte Carlo simulation (Hastings, 1970) where the number of simulated learners exposed to each of the four demonstrated knots matched the number of participants in each experimental condition (see Table 1). A value for each of the four parameters (*p*, *g*, *r*, *s*) was sampled from a uniform distribution between 0 and 1. The knot tied by each simulated learner was derived by walking through the relevant probability tree with a Bernoulli trial at each internal node (e.g. Figure 2 for a learner that observes demonstration of knot RR). This simulation procedure was repeated many times to build up parameter distributions for all the simulations that resulted in a metric value, 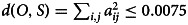, coming from fewer than 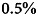 of the simulations.

Figure 5a shows uncertainty in handedness bias, with a broad distribution around a mean of 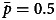 (*sd* = 0.28) which is where handedness bias is absent. The model predicts that individuals mirror fairly frequently (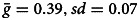) but that knots are mirrored less often than they are correctly interpreted. There is uncertainty in the posterior estimate of the repetition bias, but with a trend to be more likely to repeat the handedness of the first trefoil tied than not (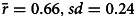). Finally, there is relatively high copying fidelity of the perceived knot (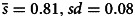).

We can establish what effect our parameter estimates would have on the cultural evolution of granny and reef knots by plugging the central tendency values into the model. For illustration, we use the mean from each posterior parameter distribution, but note that sampling from the posterior distributions each generation gives similar results (see Section S11.)

Figure 5b illustrates how the population evolves towards a single polymorphic equilibrium state, no matter the starting distribution (grey arrows leading to black disc). Compared with the case where handedness and repetition errors are random (*p* = 1/2, *r* = 1/2; blue arrows leading to blue disc), the mean posterior estimate of repetition bias results in a higher equilibrium frequency of granny over reef knots. The mean posterior estimate for the handedness coefficient is unbiased, 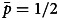, resulting in the equal equilibrium frequency of left and right forms of granny knot. As established in the social transmission model analysis, the posterior copying fidelity value, 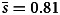 actually has no effect on the equilibrium frequencies when there is no handedness bias. The posterior mirroring value is not large enough to cause the characteristic oscillating dynamics shown in Figure 3d. We see that the equilibrium frequency results in a prevalence of granny knots over reef which can also be explored by sampling from the posterior distribution of parameter values, allowing us to see the relative frequency of granny knots over reef knots for one generation (see Section S12).

As an aside, in Section S9 we compare our social transmission model against a non-parametric estimate of equilibrium frequencies which can be found simply by iterating this proportional change without specifying the effects of specified learning processes (Claidière et al., 2014). Our parametric social transmission model results in similar equilibrium frequencies to this non-parametric prediction, indicating that the close match in the proportional change in knot frequencies over one social transmission episode caused by implementing ABC is preserved over multiple generations.

A comprehensive out-of-sample test of the model is for a future study, although we note that, for composite knots within the Ashley corpus, the proportion of granny to reef knots exactly matches the 3 to 1 ratio predicted by the equillibrium state of our model when handedness and repetition errors are random (*p* = 0.5, *r* = 0.5; blue disc in Figure 5b) and is fairly similar to that predicted using the posterior parameter estimates (mean posterior estimates give 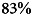 granny knots; black disc in Figure 5b; Ashley, 1993; Scanlon, 2016).

## Discussion

Our results suggest that participants exhibited a tendency for repetition and had approximately a one-in-five chance of failing to faithfully reproduce the perceived trefoil, which was sometimes the mirror image of the demonstrated knot. There was no clear handedness bias although the posterior exhibited considerable uncertainty. Our model predicts that a population expressing these posterior estimates would evolve towards an equilibrium characterised by a preponderance of granny knots over reef knots. Exploration of the model contextualises this finding to show that the prevalence of granny over reef knots is to be expected across most of parameter space, including in the absence of handedness and repetition biases. These results are consistent with empirical evidence for a prevalence of granny over reef knots found in Ashley's collection. Our results show that this pattern may not necessarily be caused by a preference for granny over reef knots, but may simply be the outcome of copying error processes affecting the construction of the knots.

ABC is a useful inductive tool to estimate the probabilistic effects of distinct putative learning processes that interact in ways specified by a social transmission model (Kandler and Powell, 2018). The model can be used both to understand how learning processes can affect cultural evolutionary dynamics and to predict evolutionary trajectories based on posterior estimates. The strong copying fidelity of the perceived knot suggests that the demonstrated knot affected participant behaviour, yet analysis of the model shows that this fidelity will not affect equilibrium frequencies in the absence of a handedness bias: there was large uncertainty over the handedness posterior estimate with only a very weak unbiased central tendency which would result in evolution towards parity of left- and right-handed knots. The effect of mirroring on evolutionary dynamics is contingent on copying fidelity of the perceived knot. Our posterior mirroring estimate suggests that faithful cultural transmission of bilaterally symmetrical tasks can be vulnerable to the correspondence problem (Heyes and Bird, 2007). Our experimental setup had learners sitting opposite the demonstrator's perspective and so our posterior mirroring value provides an estimate of the maximum mirroring effect, presuming that learners may be less vulnerable to this error if they were to sit side-by-side, taking a similar visual perspective. Nonetheless, our analysis indicates that mirroring typically has little effect on the relative equilibrium frequencies of granny and reef knots. The repetition posterior estimate exhibited considerable uncertainty but with a trend towards high values. While complex skills can be honed by repetition, a tendency for inadvertent repetition of an action can reduce within-sequence variation over cultural generations, in this case promoting granny over reef knots, and that even a small repetition bias can have a substantial effect on evolutionary dynamics within our system.

The participants’ response to the task, reflected in the posterior distributions, is likely to have been shaped by genetic and cultural influences, including experience tying either a trefoil or a composition of them. Future studies can establish the generality of these posterior estimates and the relevance of the predicted evolutionary trajectories, both for these compositions of overhand knots and for overhand knot structures within more complex knots. Similarity between the equilibrium state predictions and the relative frequencies of granny and reef knots in the Ashley corpus provides some support for the model's out-of-sample performance, although it is not clear that Ashley's depiction of handedness in composite knot forms accurately reflects their relative frequencies in the wild. Nonetheless, our model helps to explain the apparent prevalence of granny over reef knots when functional investigation suggests that the reef knot is superior (Grog, n.d.; O'Reilly et al., 2017).

Under some conditions, the model behaviour contradicts the common assertion that population-level homogeneity is a signature of high copy fidelity. If there is a handedness bias or propensity for repetition, our system exhibits greater homogeneity (a preponderance of granny knots) at equilibrium when copying fidelity is low than when it is high: low copying fidelity allows the handedness and repetition to take effect, reducing heterogeneity. Thus population-level measures of cultural variation are not necessarily accurate proxies of between-individual learning processes (Acerbi et al. 2016; but see Smaldino et al. 2018 and Acerbi et al. 2018). Copying fidelity in our model is of the demonstrated knot as perceived by the learner and so a copying fidelity of *s* = 1 can still result in an error if there is mirroring. Nonetheless, when there is a bias in handedness or a propensity for repetition, the relationship between copying fidelity and heterogeneity at equilibrium holds as mirroring typically has little effect the relative equilibrium frequencies of granny and reef knots.

More generally, imperfect copying encourages the evolution of heterogeneity when there is a closed set of alternative behaviours and failure to copy one variant results in adoption of another (see Section S10). This mechanism is responsible for the model's prediction that the two reef knot forms will evolve towards equal frequencies; note there is no assumption that individuals recognise both forms of reef knot to be mathematically indistinct. Mirroring also pushes the population toward equal frequencies of knot forms because it is most likely, by chance, to reverse the handedness of the most common trefoil.

Future work can explore conditional relations between the learning processes by comparing explanatory value of alternative putative conditional rules through model selection. Rules such as copy the perceived trefoil only if there is no handedness bias would would require a new model as, for each trefoil, the copying parameter *s* lies on a different branch of the tree from the absence of handedness effect (1 − *p*) (see Figure 2). It will also be valuable to incorporate effects of perceived functionality and social value associated with knot structures in future analysis. Evolvability of complex knots within design space may be particularly susceptible to copying error biases where variation in knot structure is redundant in relation to practical or social function.

Commenting on the utility of evolutionary approaches to study patterns of artefact variation, Lycett (2015, p. 27) states that ‘some of the most key advances in evolutionary approaches over the coming years are likely to center on increased empirical understanding of the links between processes of transmission and resultant artefactual variation, and moreover, the types of behavioural factors that influence patterns of variation in particular ways’. To this end, our study provides statistical evidence that putative learning processes interact to affect cultural evolutionary dynamics of bilaterally-symmetrical artefact production. Our experimental and theoretical simulations of social transmission can be complimented both by controlled experiments to uncover proximal cognitive mechanisms underpinning the identified statistical profile, and by ethnographic accounts of social and functional value including group identity, aesthetic appeal and pedagogical norms scaffolding transmission.

## Supplementary material

For supplementary material accompanying this paper visit http://dx.doi.org/10.1017/ehs.2019.17. click here to view supplementary material

## Data accessibility

Data are available from the Open Science Framework: https://doi.org/10.17605/OSF.IO/GZ2PS
