# Supplementary material for: Unknotting the interactive effects of learning processes on cultural evolutionary dynamics
Source: Evol Hum Sci. 2019 Dec 23;1:e17. doi: 10.1017/ehs.2019.17 (PMC10427294; doi:10.1017/ehs.2019.17)
Supplement: Supplementary file 1 [file S2513843X19000173sup001.pdf]

SUPPLEMENTARY MATERIAL: UNKNOTTING THE INTERACTIVE EFFECTS OF LEARNING PROCESSES ON CULTURAL EVOLUTIONARY DYNAMICS

LAUREN A. SCANLON[1,2], ANDREW LOBB[1], JAMSHID J. TEHRANI[2], JEREMY R. KENDAL[2]
[1]DEPARTMENT OF MATHEMATICS, DURHAM UNIVERSITY
[2]DEPARTMENT OF ANTHROPOLOGY AND DURHAM CULTURAL EVOLUTION RESEARCH CENTRE, DURHAM UNIVERSITY
CORRESPONDENCE EMAIL: JEREMY.KENDAL@DURHAM.AC.UK

S1. Knot Invariants

Knot invariants will give the same result when two knots are the same, and different results when they are distinct. The Jones polynomial Jones (1985), denoted $V(t)$, is one such invariant, which assigns a Laurent polynomial with integer coefficients in one variable $t^{1/2}$ to each knot and gives us some information about the crossings of that knot. The Jones polynomial is given for the left-handed granny knot in Equation S1, the right-handed granny knot in Equation S2 and both reef knots by Equation S3. We can see that the Jones polynomial for the left-handed granny knot differs from the right-handed granny knot by the sign of the exponents in the polynomial, the exponents for the left-handed granny knot are all negative whilst they are positive for the right-handed granny knot. This is the only difference between the two polynomials and shows that the left-handed and right-handed granny knot are mirror images of one another. Both versions of the reef knot have the same Jones polynomial which contains both positive and negative values for the exponents showing that there is no difference between the two versions of this knot. These polynomials show that the granny knots are distinct from each other and both reef knots, but the two reef knots are not distinct, which can be seen by rotating one reef knot to match the other; no such rotation is possible for the granny knots (See Figure 1c).

$$V_{LL}(t) = t^{-2} + 2t^{-4} - 2t^{-5} + t^{-6} - 2t^{-7} + t^{-8} \tag{S1}$$

$$V_{RR}(t) = t^{2} + 2t^{4} - 2t^{5} + t^{6} - 2t^{7} + t^{8} \tag{S2}$$

$$V_{reef}(t) = -t^{3} + t^{2} - t + 3 - t^{-1} + t^{-2} - t^{-3} \tag{S3}$$

S2. Asocial Handedness Bias Experiment

We asked participants to tie a "simple knot". We then checked that this was a trefoil knot. The knot was undone, then participants were asked to tie a "simple knot" every 60s over a 10 minute period. Each knot was tied in a separate 25cm length of string and the sealed in a small plastic bag. Over the same period, participants were asked to complete a distraction task in between tying each knot, requiring them to draw six concepts in order that another person could match the concepts to the drawings at a later time. Both the plastic bag containing the 10 knots and the paper with the drawings from the distraction task were collected in at the end of this stage.

For each participant, we recorded knot handedness over the 10 trefoils as an estimate of knot handedness bias in the absence of a demonstration. The frequency of right-handed trefoils tied by each person is shown in Figure S1, where participants who tied no right-handed trefoils tied all left-handed trefoils. Two participants tied knots that were not trefoils and have not been included in these data.

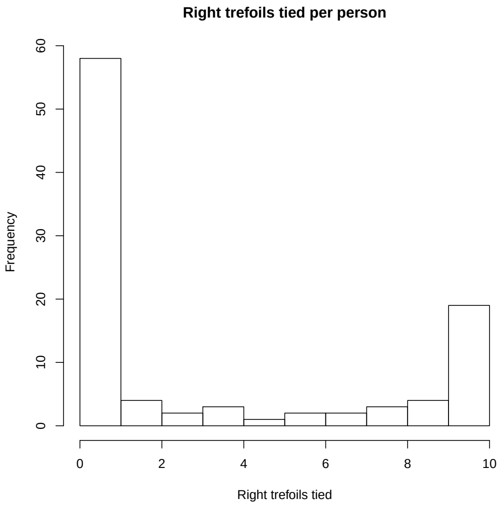

Figure S1. Frequency of right-handed trefoils tied by participants, those who tied no right-handed tied all left-handed trefoils and vice versa

The majority of participants tied either all right-handed or all left-handed trefoils, with a few tying a mixture of the two. Left-handed trefoils were much more common than right-handed trefoils. The mean proportion of right-handed trefoils

tied per person was 0.32. This asocial handedness bias is compared against the handedness bias estimate derived from the social transmission experiment. See Section S4) for weak evidence that individuals who typically write with their right hand were more likely to tie a left-handed trefoil than those who write with their left, while those using their left hand to write were more likely to tie a right-handed trefoil than those who use their right. This weak evidence agrees with Chisnall (2010) who, through a survey involving the tying of multiple knots including trefoil knots and shoelace knots, found right handers tied a higher proportion of left handed knots than left handers and visa versa.

We note some association between the asocial handedness bias and the first knot tied by participants in the social transmission experiment (Table S1).

		Social trans. expt. knots tied				
		LL	RR	LR	RL	Total
Asocial handedness bias	Left	25	20	12	11	68
	Right	6	9	2	12	29
	Total	31	29	14	23	97

Table S1. Knot frequencies in the social transmission experiment given handedness of trefoil previously tied by the same participants under asocial conditions; dashed lines delineate granny knots from reef knots.

S3. Questionnaire Information

Participants were asked to complete a questionnaire detailing their name, gender, degree programme, handedness and hand usually written with and whether they knew how to tie a reef or granny knot. The questionnaire was filled in by participants at the end of the experiment, when all materials had been collected.

		Trefoil Tied		
		Right	Left	Total
Hand usually written with	Right	25	62	87
	Left	4	6	10
Self-reported handedness	Right	23	58	81
	Left	4	5	9
	Ambidextrous	2	5	7
	Total	29	68	97

Table S2. Handedness of trefoils tied given hand usually written with.

The majority of participants usually wrote with their right hand and tied a majority of left-handed trefoils. Using Bayesian association analysis (Gelman et al., 2003; Bååth, 2014) shown in Figure S2 we see there is weak evidence for a larger probability of tying a left-handed trefoil than right-handed trefoil by participants who usually wrote with their right hand than those who wrote with their left. Similarly there is weak evidence for a larger probability of tying a right-handed trefoil than left-handed trefoil by those who usually wrote with their left hand. However, the proportion of participants who usually wrote with their left hand is quite low so might not be wholly representative. A similar result can be found using the self reported handedness data with those reporting as ambidextrous having a larger probability of tying a left- than a right-handed trefoil. Acknowledging the small sample size, most of those reporting as ambidextrous usually wrote with their right hand which fits with the test of proportions for hand written with and trefoil tied.

Participants were asked to record their gender in a free-form box.

		Tied correct knot		
		Y	N	Total
Gender	Male	19	17	36
	Female	28	33	61
	Other	2	1	3
	Total	49	51	100

Table S3. Performance in experiment given gender

Table S3 shows the proportion of participants who tied the knot shown in the video given their gender. It is clear to see that gender had no bearing on their performance in the experiment.

Participants were also asked whether they knew how to tie a granny and a reef knot.

		Knot tied		
		Granny	Reef	Total
Knew how to tie a granny knot	Yes	17	13	30
	No	45	25	70
	Total	62	38	100

Table S4. Performance in experiment given knowledge of granny knots

		Knot tied		
		Granny	Reef	Total
Knew how to tie a reef knot	Yes	17	17	34
	No	45	21	66
	Total	62	38	100

Table S5. Performance in experiment given knowledge of reef knots

Tables S4 and S5 show the proportion of participants who tied granny and reef knots given the self-reported knowledge. Approximately one third of participants reported that they knew how to tie each knot. There is weak evidence that overall bias towards granny knots over reef knots is stronger in those that self-reported that they did not know how to tie these knots than those that did.

S4. Association Analysis

Posterior simulations of the test of proportions generated using R package Bayesian First Aid (Bååth, 2014). The test of proportions assumes flat priors constructed as a Beta(1,1) distribution.

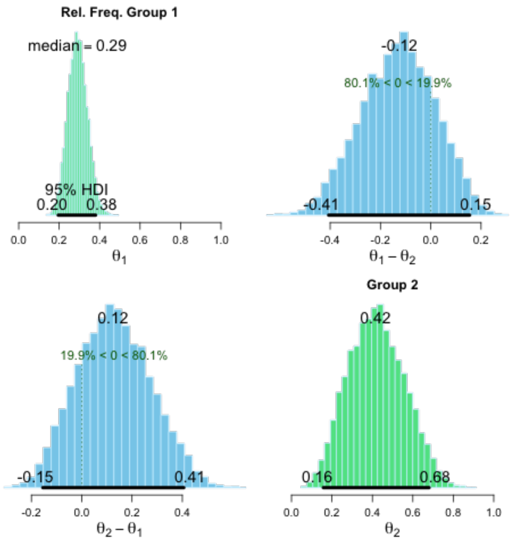

(a) Posterior simulation of right trefoils tied

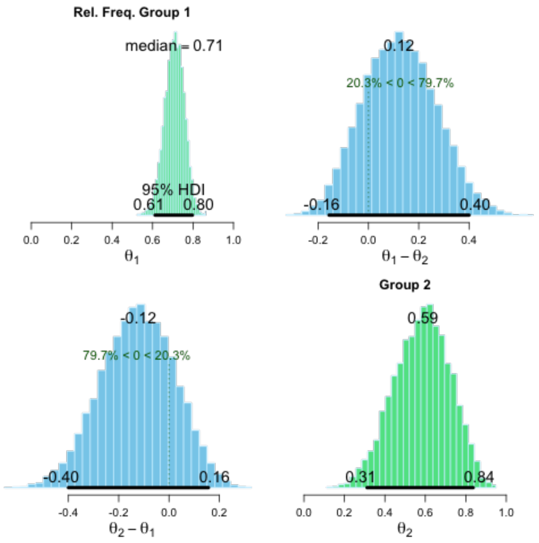

(b) Posterior simulation of left trefoils tied

Figure S2. Figure S2a shows the posterior simulations of tying a right handed trefoil by those who wrote with a specified hand. θ_1 refers to those who wrote with their right hand and tied a right trefoil whilst θ_2 refers to those who wrote with their left hand and tied a right trefoil. The differences $\theta_1 - \theta_2$ and $\theta_2 - \theta_1$ refer to the difference between these groups. There is weak evidence that a larger probability of those who write with their left hand tie a right handed trefoil than those who wrote with their right hand. Figure S2b shows the posterior simulations of tying left handed trefoils by those who wrote with either hand. θ_1 refers to those who wrote with their right hand and tied a left trefoil whilst θ_2 refers to those who wrote with their left hand and tied a left trefoil. The differences $\theta_1 - \theta_2$ and $\theta_2 - \theta_1$ refer to the difference between these groups. There is weak evidence that a larger probability of those who write with their right hand tie a left handed trefoil than those who wrote with their left hand. If we look at both Figures S2a and S2b we see those who wrote with their right hand were more likely to tie a left- than a right-handed trefoil. Those who wrote with their left hand were slightly more likely to tie a left handed trefoil than a right handed as the left handed trefoil was the most common amongst both groups and there were relatively few people reporting as writing with their left hand.

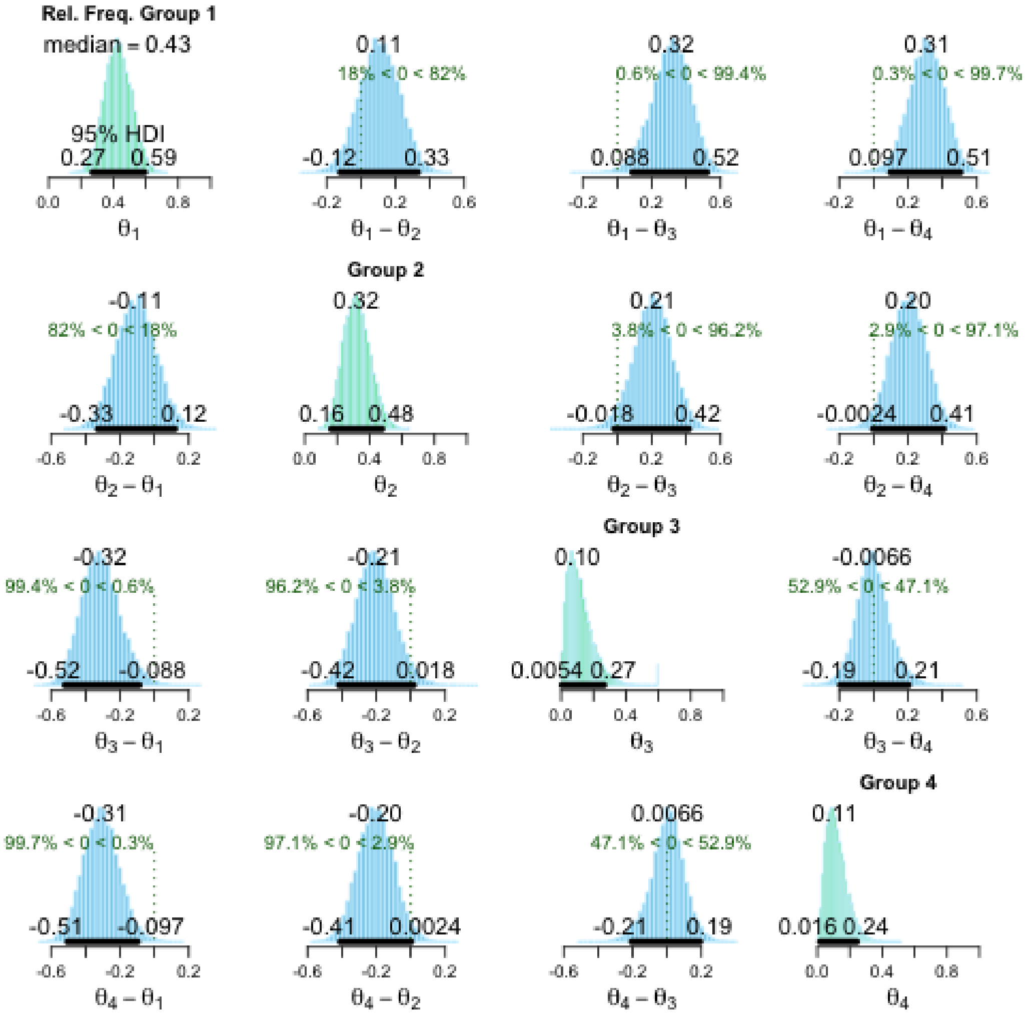

Figure S3. Posterior simulation of LL knots tied given demonstration knot. θ_1 refers to those who were shown the knot LL and tied LL, θ_2 those who were shown RR and tied LL, θ_3 those who were shown LR and tied LL and θ_4 those who were shown RL and tied LL with $\theta_i - \theta_j, (i, j \in \{1, 2, 3, 4\}, i \neq j)$ referring to the difference between groups. We see a larger probability for those who were shown either LL or RR tying LL than LR or RL, with those shown LL having the largest probability.

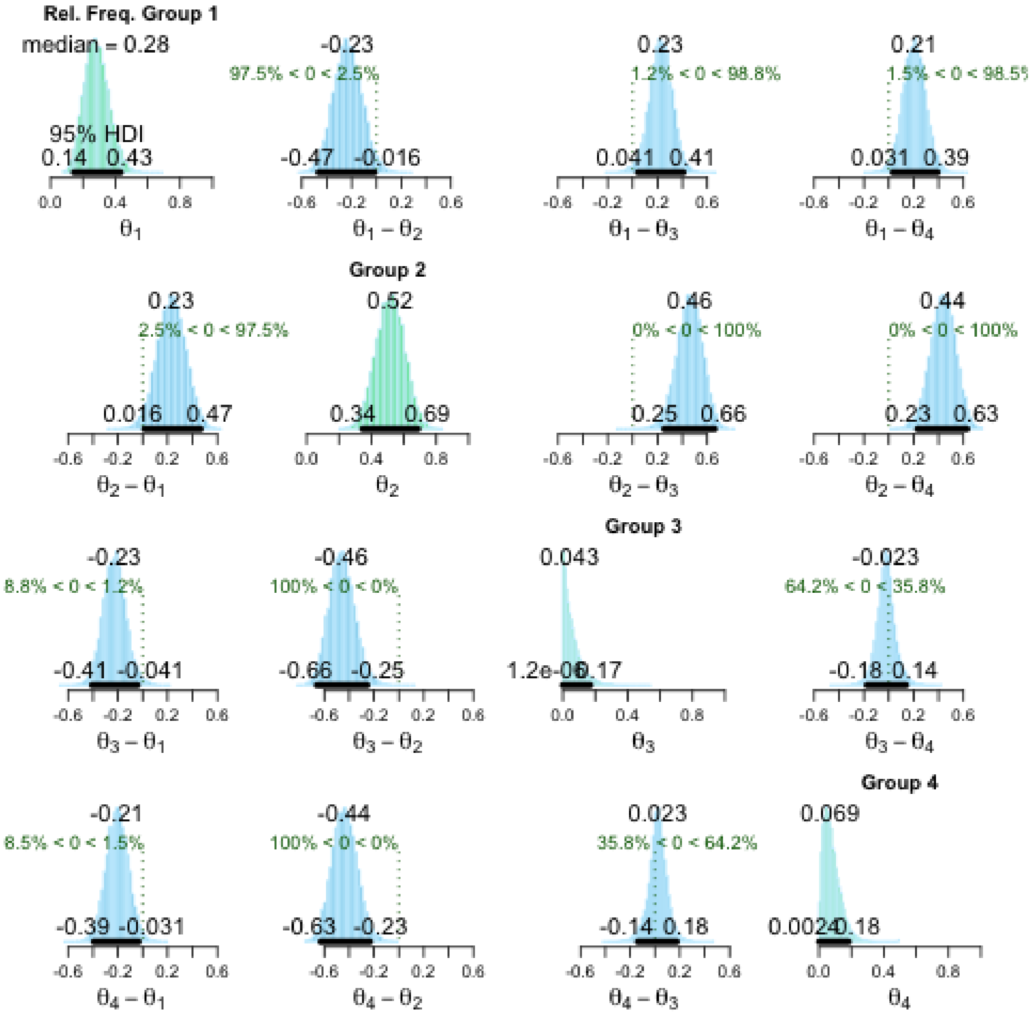

Figure S4. Posterior simulation of RR knots tied given demonstration knot. θ_1 refers to those who were shown the knot LL and tied RR, θ_2 those who were shown RR and tied RR, θ_3 those who were shown LR and tied RR and θ_4 those who were shown RL and tied RR with $\theta_i - \theta_j, (i, j \in \{1, 2, 3, 4\}, i \neq j)$ referring to the difference between groups. We see a larger probability for those who were shown either LL or RR tying RR than LR or RL, with those shown RR having the largest probability.

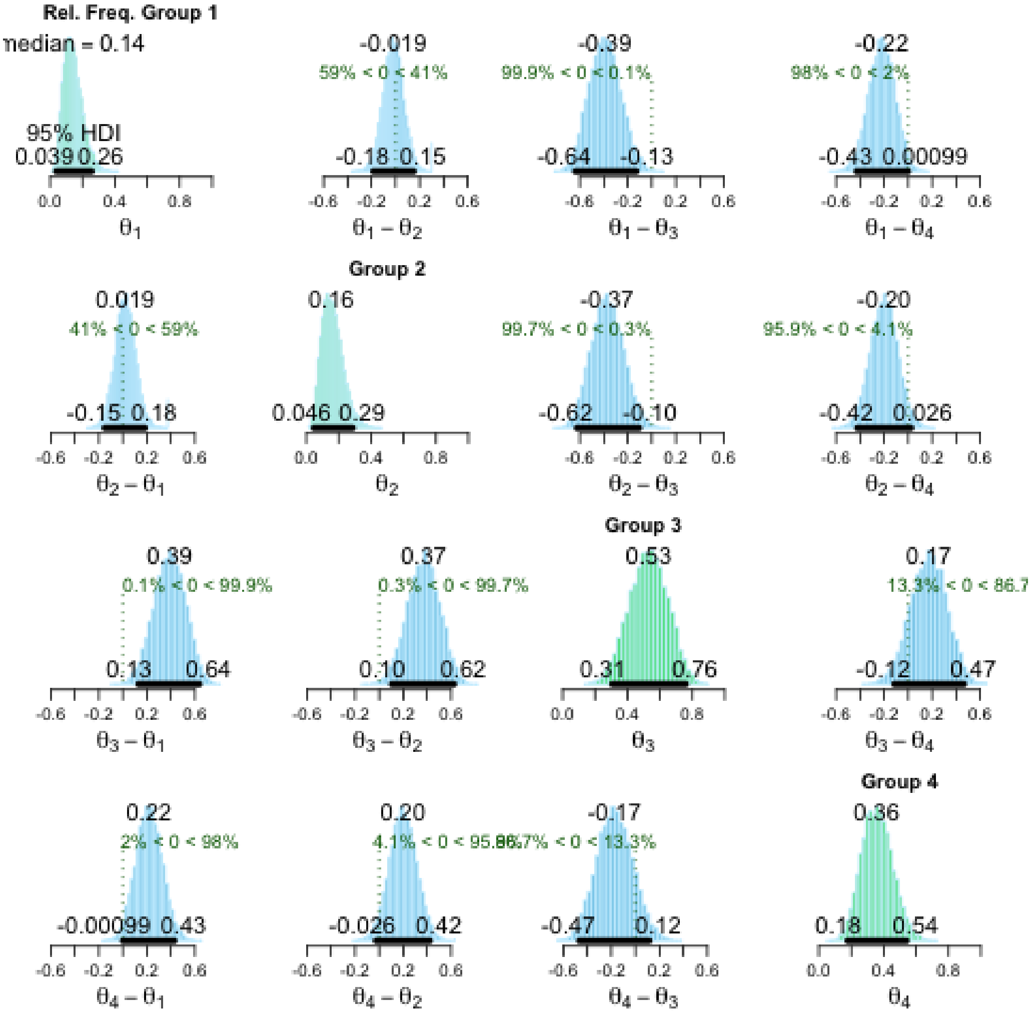

Figure S5. Posterior simulation of LR knots tied given demonstration knot. θ_1 refers to those who were shown the knot LL and tied LR, θ_2 those who were shown RR and tied LR, θ_3 those who were shown LR and tied LR and θ_4 those who were shown RL and tied LR with $\theta_i - \theta_j, (i, j \in \{1, 2, 3, 4\}, i \neq j)$ referring to the difference between groups. We see a larger probability for those who were shown either LR or RL tying LR than LL or RR, with those shown LR having the largest probability.

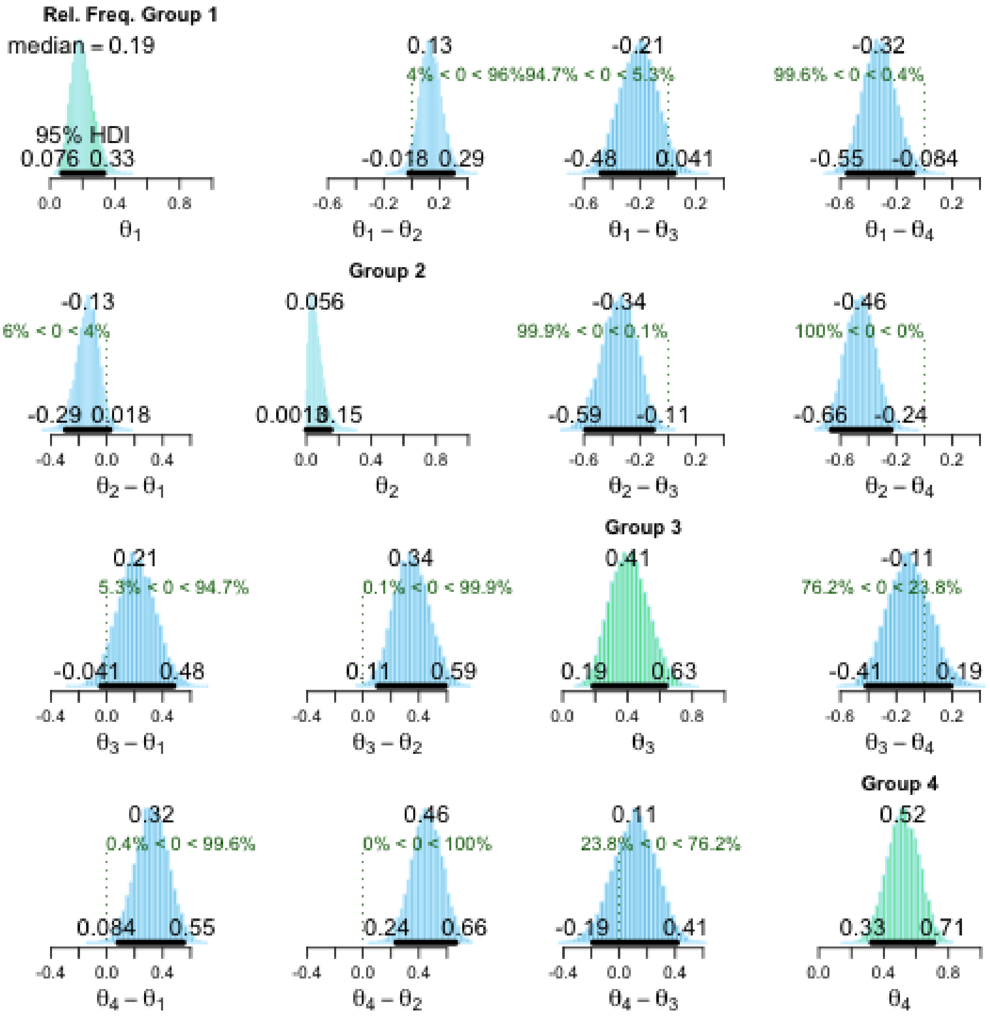

Figure S6. Posterior simulation of RL knots tied given demonstration knot. θ_1 refers to those who were shown the knot LL and tied RL, θ_2 those who were shown RR and tied RL, θ_3 those who were shown LR and tied RL and θ_4 those who were shown RL and tied RL with $\theta_i - \theta_j, (i, j \in \{1, 2, 3, 4\}, i \neq j)$ referring to the difference between groups. We see a larger probability for those who were shown either LR or RL tying RL than LL or RR, with those shown RL having the largest probability.

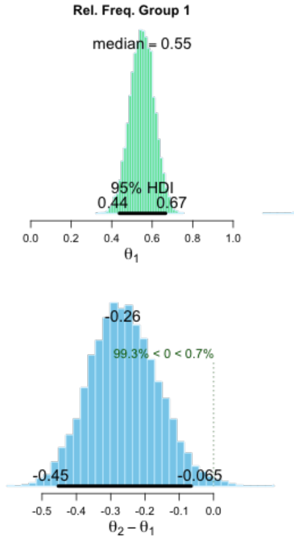

(a) Posterior simulation of knots tied by those with a left hand bias when tested under asocial conditions

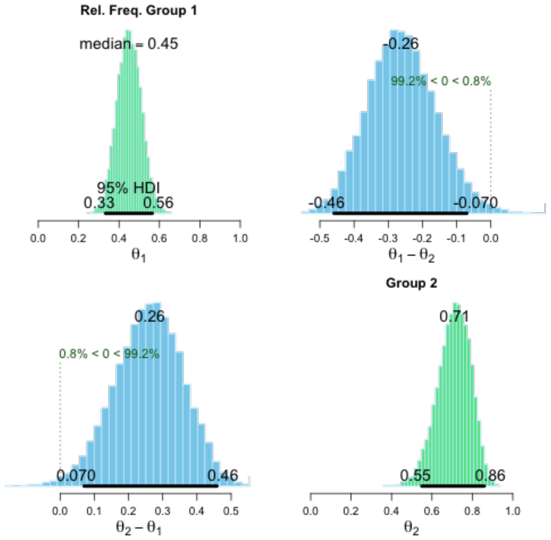

(b) Posterior simulation of knots tied by those with a right hand bias when tested under asocial conditions

Figure S7. Posterior simulations of first tying an L or R knot following demonstration given a left-hand bias under asocial conditions. θ_1 refers to those who had a left hand bias under asocial conditions and tied an L knot first following demonstration, θ_2 those who had a left hand bias and tied an R knot first and $\theta_1 - \theta_2$ and $\theta_2 - \theta_1$ the difference between groups. We see there is a larger probability of those who had a left hand bias starting their post-demonstration knot with an L knot than an R. Figure S7b shows the simulations of tying an L or R knot first following demonstration given a right hand bias under asocial conditions. θ_1 refers to those who had a right hand bias under asocial conditions and tied an L knot first following demonstration, θ_2 those who had a right hand bias and tied an R knot first and $\theta_1 - \theta_2$ and $\theta_2 - \theta_1$ the difference between groups. We see there is a larger probability of those who had a right hand bias starting their post-demonstration knot with an R knot than an L.

S5. Recursion Equations

The equations are

$$
\begin{aligned}
f'_{RR} =& f_{RR}((1-g)s^2 + (1-s)^2(1-r)p^2 + (1-s)^2rp + 2(1-g)s(1-s)r) \\
&+2(1-g)s(1-s)(1-r)p \\
&+f_{LL}((1-s)^2(1-r)p^2 + (1-s)^2rp + gs^2 + 2gs(1-s)r) \\
&+2gs(1-s)(1-r)p \\
&+(f_{RL} + f_{LR})((1-s)^2(1-r)p^2 + (1-s)^2rp + s(1-s)r \\
&+s(1-s)(1-r)p)
\end{aligned} \tag{S4}
$$

$$
\begin{aligned}
f'_{LL} =& f_{RR}(gs^2 + (1-s)^2(1-r)(1-p)^2 + (1-s)^2r(1-p) + 2gs(1-s)r \\
&+2gs(1-s)(1-r)(1-p)) \\
&+f_{LL}((1-g)s^2 + (1-s)^2(1-r)(1-p)^2 + (1-s)^2r(1-p) \\
&+2(1-g)s(1-s)(1-r)(1-p) + 2(1-g)s(1-s)r) \\
&+(f_{RL} + f_{LR})((1-s)^2(1-r)(1-p)^2 + (1-s)^2r(1-p) \\
&+s(1-s)(1-r)(1-p) + s(1-s)r)
\end{aligned} \tag{S5}
$$

$$
\begin{aligned}
f'_{RL} =& f_{RR}((1-s)^2(1-r)p(1-p) + (1-g)s(1-s)(1-r)(1-p) \\
&+g(1-s)s(1-r)p) \\
&+f_{LL}((1-s)^2(1-r)p(1-p) + (1-g)(1-s)s(1-r)p \\
&+gs(1-s)(1-r)(1-p)) \\
&+f_{RL}((1-g)s^2 + (1-s)^2(1-r)p(1-p) + (1-g)s(1-s)(1-r)) \\
&+f_{LR}(gs^2 + (1-s)^2(1-r)p(1-p) + gs(1-s)(1-r))
\end{aligned} \tag{S6}
$$

$$
\begin{aligned}
f'_{LR} =& f_{RR}((1-s)^2(1-r)(1-p)p + (1-g)(1-s)s(1-r)(1-p) \\
&+gs(1-s)(1-r)p) \\
&+f_{LL}((1-s)^2(1-r)(1-p)p + (1-g)s(1-s)(1-r)p \\
&+g(1-s)s(1-r)(1-p)) \\
&+f_{RL}(gs^2 + (1-s)^2(1-r)(1-p)p + gs(1-s)(1-r)) \\
&+f_{LR}((1-g)s^2 + (1-s)^2(1-r)(1-p)p + (1-g)s(1-s)(1-r))
\end{aligned} \tag{S7}
$$

S6. Equilibria Equations

Equilibria occur when

$$\hat{f}_{RR} = \frac{Q_1}{P}$$

where

(S8)

$$Q_1 = -p^2(r-1)(s-1)(1+s(2g-1)(r-1)+rs^2(2g-1)) + gs(r(s^2-2)-s) \\ + p(s-1)(2gs + r^2 s(2g-1)(1+s) + r(1+s-2gs(2-s)))$$

$$\hat{f}_{LL} = \frac{Q_2}{P}$$

where

$$\text{(S9)} \quad Q_2 = s^2(1-g) - p^2(r-1)(s-1)(1+s(2g-1)(r-1)+rs^2(2g-1)) - 1 \\ + r(s(1-2g)+s^3(g-1)) + p(s-1)(r^2 s(2g-1)(1+s) \\ + 2s(g-1) + rs(1+(3-4g)-2s^2(g-1)) - 2)$$

$$\hat{f}_{LR} = \frac{Q_3}{P}$$

where

$$\text{(S10)} \quad Q_3 = (r-1)(gs - p(s-1)(1+p^2(s-1))(1+(2g-1)(s(r-1)+rs^2)))$$

$$\hat{f}_{RL} = \frac{Q_4}{P}$$

where

$$\text{(S11)} \quad Q_4 = (r-1)(gs - p(s-1)(1+p^2(s-1))(1+(2g-1)(s(r-1)+rs^2)))$$

and

$$\text{(S12)} \qquad P = (1+s)(s(2g-1)(rs-r-1)-1).$$

S7. Stability

In this system, an equilibrium point is stable if no matter the starting values of f_{RR}, f_{LL}, f_{LR}, f_{RL}, the system comes to rest at the same point. If the point changes depending on these starting values then it is not stable.

To find the stable equilibrium points we set f_{ij} equal to the equilibria points determined by the equations, plus some small perturbation ϵ_{ij}. The equilibrium is stable if the value of f'_{ij}, moves towards the equilibria points given by the equations in Appendix S6.

Let

$$f_{RR} = \frac{Q_1}{P} + \epsilon_{RR} \tag{S13}$$

$$f_{LL} = \frac{Q_2}{P} + \epsilon_{LL} \tag{S14}$$

$$f_{LR} = \frac{Q_3}{P} + \epsilon_{LR} \tag{S15}$$

$$f_{RL} = \frac{Q_4}{P} + \epsilon_{RL} \tag{S16}$$

where Q_i and P are as given in Appendix S6, and

$$\epsilon_{RL} = -\epsilon_{RR} - \epsilon_{LL} - \epsilon_{LR} \tag{S17}$$

to ensure f_{ij} sum to one.

We then compute f'_{RR}, f'_{LL}, f'_{LR}, f'_{RL} and the distance:

$$d_{RR} = f'_{RR} - \frac{Q_1}{P} \tag{S18}$$

$$d_{LL} = f'_{LL} - \frac{Q_2}{P} \tag{S19}$$

$$d_{LR} = f'_{LR} - \frac{Q_3}{P} \tag{S20}$$

$$d_{RL} = f'_{RL} - \frac{Q_4}{P} \tag{S21}$$

We then have the following cases.

Case 1:

$$d_{ij} = 0 \tag{S22}$$

In this case the system jumps to an equilibrium point given by the parameters. The system then remains at this point for all generations. This occurs when $s = 0$. The system is not affected by starting values of f_{ij}, the frequency of each type of knot is determined solely by the values of p and r.

Case 2:

$$d_{ij} = \epsilon_{ij} \tag{S23}$$

In this case there is no change in the system, meaning the system is currently at equilibria, with the system remaining at this point for all generations. This occurs when copying is always accurate and mirroring never occurs, when $s = 1$ and $g = 0$. The equilibrium state is determined by the starting values of f_{ij} and is independent of the values of p and r. The frequency of each type of knot remains constant across generations.

Case 3:

$$d_{ij} < \epsilon_{ij} \tag{S24}$$

In this case the system moves towards the equilibrium point given by the parameters. This occurs when $s < 1$ and the system evolves towards equilibria over generations.

Case 4:

$$d_{ij} > \epsilon_{ij} \tag{S25}$$

In this case the system moves away from the equilibrium point given by the parameters. This never occurs for any equilibrium point in the system, meaning all points are stable.

S8. Barycentric Coordinates

We plot a tetrahedron with vertices at the points $\begin{pmatrix}1\\0\\0\end{pmatrix}$, $\begin{pmatrix}0\\1\\0\end{pmatrix}$, $\begin{pmatrix}0\\0\\1\end{pmatrix}$ and $\begin{pmatrix}1\\1\\1\end{pmatrix}$.

Taking values of f'_{ij} from our equations, we can represent the values of f'_{ij} as points $\mathbf{p}$ inside the tetrahedron using the conversion

$$\mathbf{p} = \begin{pmatrix} f'_{RR} + f'_{RL} \\ f'_{LL} + f'_{RL} \\ f'_{LR} + f'_{RL} \end{pmatrix} \tag{S26}$$

S9. Non-parametric Estimate of Equilibrium State

Following Claidière et al. (2014), we construct a transmission matrix taken directly from the experimental data (Table 1), which represents the probability of the change in knot types from those demonstrated to those learned. For example $x_{2,1} = P(LL|RR)$ is the probability of tying knot LL when shown RR.

$$X = \begin{bmatrix} \frac{14}{26} & \frac{9}{26} & \frac{1}{26} & \frac{2}{26} \\ \frac{9}{25} & \frac{15}{25} & 0 & \frac{1}{25} \\ \frac{4}{24} & \frac{4}{24} & \frac{8}{24} & \frac{8}{24} \\ \frac{6}{25} & \frac{1}{25} & \frac{6}{25} & \frac{12}{25} \end{bmatrix} \tag{S27}$$

X is a right stochastic matrix representing the frequency of change in knots tied given by the experimental data. We can simulate social transmission of these knots within future generations by taking powers of this matrix, basing future generations solely on the present state. This approach treats any parameters affecting change in cultural variant frequency as implicit, linear effects in the transition matrix. After 20 generations we have stability in transmission such that the probability of tying any given knot remains constant (measured to 3 decimal places).

Knot	Parametric	Non-parametric
LL	41.5%	40.1%
RR	41.5%	39.1%
LR	8.5%	7.2%
RL	8.5%	13.6%

Table S6. Percentage of each type of knot at equilibrium predicted by the parametric social transmission model, using ABC-derived mean posterior parameter values, and the non-parametric approach.

Table S6 shows that both the parametric and non-parametric models predict a prevalence of granny over reef knots at equilibrium, but unlike the non-parametric approach, the parametric social transmission model gives equal frequencies of both reef knots. The non-parametric approach makes no theoretical assumptions over how copying fidelity, mirroring, repetition and handedness bias interact so it is unsurprising to find unequal reef knot frequencies. The parametric model behaviour is, by definition, determined by the probabilistic interactions of (s, g, r, p) but the model does not assume that individuals recognise or treat the two reef knots to

be mathematically the same. The similarity in the predictions between the parametric and non-parametric approaches indicates that the ABC-derived parameter estimates do a good job at estimating the steady state frequencies derived, using the transition matrix, by the experiment data alone.

S10. Closed System Model

Consider n variants, each of which occurs at frequency f_i, where $\sum_{i=1}^{n} f_i = 1$. Frequencies in the subsequent cultural generation, f', are determined by oblique transmission with copying fidelity s, where failure to copy variant i results in randomly adopting one of the $n-1$ other variants;

$$f_i' = sf_i + (1-s)\frac{(1-f_i)}{n-1}. \tag{S28}$$

The equilibrium frequency $\hat{f}_i = \frac{1}{n}$.

S11. Equilibrium Distribution Given Sampled Parameter Values

The usage of the mean posterior values in Figure 5b results in the grey arrow's smooth evolutionary trajectory. This gives the assumption that the parameter values are constant for each generation, however given the distribution of parameter values seen in Figure 5a it may be more accurate to the sample from that distribution to simulate evolutionary frequencies each generation. Taking parameter values in this way, the result gives evolutionary frequencies distributed around the values resulting from taking the mean posterior values as constant parameter values for each generation, as can be seen in Figure S8.

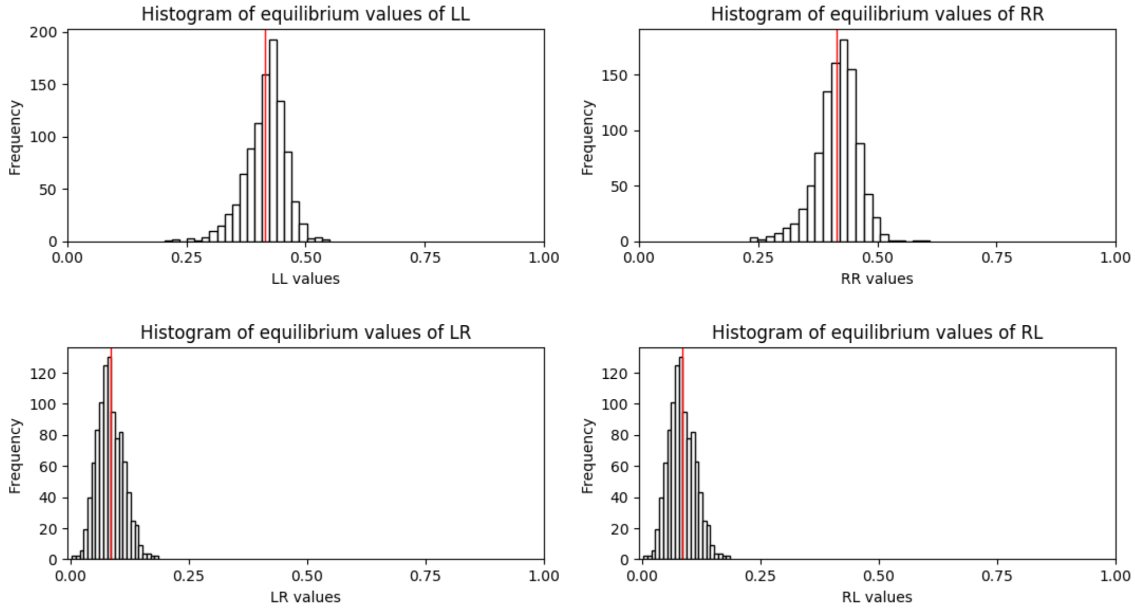

Figure S8. Equilibrium values of LL, RR, LR and RL determined by sampling from the distribution of parameter values. The red lines on each plot denote the equilibrium values determined by taking mean parameter values constant over generations

S12. Knots Frequencies After One Generation Given Sampled Parameter Values

The equilibrium frequencies in Figure 5b demonstrate the prevalence of granny knots over reef knots in the population when simulated over generations, but sampling from the posterior distribution for the parameters p, g, r and s allows us to explore the relative occurrence of each knot in one generation. Sampling from the posterior of parameter values in a way that models the experiment gives the frequency of each type of knot. In Figures S9a and S9b we show the frequency of each knot type over repeated simulations with the maximum occurrence for each knot being 25 to represent the demonstrations in the experiment. We see that both granny knots, RR and LL, occur much more frequently than the reef knots LR and RL.

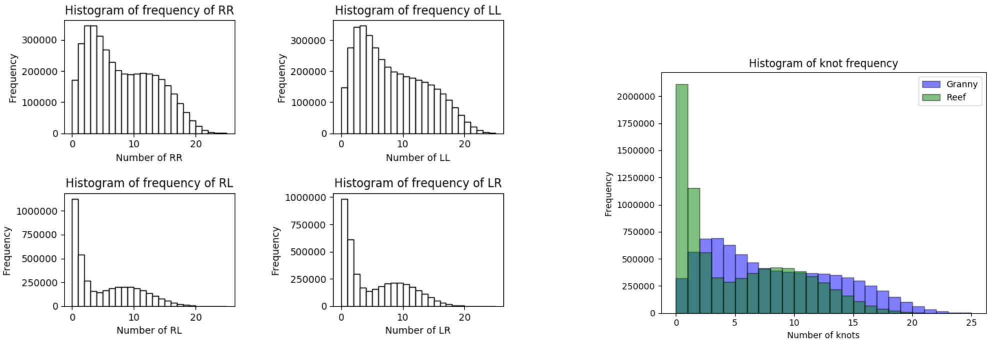

(a) (b)

Figure S9. Part (a) shows the frequencies of LL, RR, LR and RL after one generation determined by sampling parameter values from the posterior distribution. We see that this results in higher occurrences of the knots RR and LL than RL and LR. Part (b) shows the frequencies of granny and reef knots after one generation determined by sampling parameter values from the posterior distribution. We see that this results in higher occurrences of the knots granny knots over the reef with the frequency of each type of knot overlayed.

References

Bååth, R. (2014), Bayesian First Aid: A Package that Implements Bayesian Alternatives to the Classical * .test Functions in R, *in* 'UseR! 2014 - Int. R User Conf.'.

Chisnall, R. C. (2010), 'Knot-tying habits, tier handedness, and experience', *J. Forensic Sci.* **55**(5), 1232–1244.

Claidière, N., Scott-Phillips, T. C. and Sperber, D. (2014), 'How Darwinian is cultural evolution?', *Philos. Trans. R. Soc. B Biol. Sci.* (March).

Gelman, A., Carlin, J. B., Stern, H. S. and Rubin, D. B. (2003), *Bayesian Data Analysis*, Vol. 2, 3 edn, Chapman and Hall/CRC.

Jones, V. F. R. (1985), 'A polynomial invariant for knots via Von Neumann algebras', *Bull. Am. Math. Soc.* **12**(1), 103–111.
